# Surgical Observations on Tumours; with Cases and Operations

**Published:** 1838-04

**Authors:** 


### Art. VI.

*Surgical Observations on Tumours; with Cases and Operations.* By John C. Warren, m.d., Professor of Anatomy and Surgery in Harvard University, and Surgeon of the Massachusetts General Hospital.—*Boston*, 1837. 8vo. pp. 607.

Nothing is more apt to produce a favorable impression of any literary production than to find that the author has justly appreciated his own labours, and has offered them to the public under an appropriate title, and without immoderate pretensions. A prepossession is thus created in favour of his good sense, and the reader is prepared to listen with attention to one who is able to decide fairly in a matter in which the natural bias of self-approbation is frequently too strong for sober judgment. In the work before us, these first favorable impressions do not lead to disappointment. "It is not," we are told, "to be considered as a treatise comprehending all that is known on the subject, but rather as a collection of cases intended to illustrate the distinctions between different tumours." (*Pref.*, p. vi.) Now, although a collection of this kind falls short, both in the labour of compilation and in the information conveyed, of those completer works which trace out and explain in a systematic narrative the beginning, the progress, and termination of a class of disorders, it nevertheless possesses great utility, is an easy and natural mode of delivering the results of observation, and is sanctioned by the authority and example of the best practical writers of our country.

The cases which we find reported in modern medical writings are chiefly of two kinds: the one, formed on the model of the French school, and carried to the highest degree of perfection by Professor Louis, consists of a collection of the most minute particulars, whether connected or unconnected with the principal disorder; the other, which seems especially to flourish in English soil, characterized by a selection of the more prominent features which distinguish the disease, and which appear to afford some direct clue to the means of palliation or of cure. The former method, from which all hypotheses and inferences are professedly excluded, is no doubt highly philosophical, and capable, when carried out with the zeal and talents which have been bestowed upon it, of eliciting valuable truths; the latter, by its more direct tendency to practical conclusions, and the fixing of the attention on those points on which questions of interest appear to turn, is more attractive to minds accustomed to reason while they observe, and make utility and applicable knowledge the prime objects of attainment. To this last class belong the cases contained in the present volume: clear, simple, and graphic, they bear the unaffected impress of truth, and report with manifest candour and honesty, as medical writings ought, the opinions that influenced the treatment and the motives that guided the surgeon in performing or abstaining from operations. In fact, throughout his work the author proves himself to be a worthy disciple of the school in which he received his early instructions, and which, connected as it is with the names and celebrity of Cooper, Bright, and others of hardly less eminence, he seems proud to acknowledge as his alma mater: and surely that school need be no less proud to claim him as a pupil; as we believe there are few members of our profession who are more entitled to the consideration of his brethren than Dr. Warren. Distinguished as a zealous and successful author and teacher for the last thirty years, foremost in every undertaking calculated to improve medical literature and science, and never weary in the cause of benevolence, he has long earned for himself, in his native country, a reputation of the most enviable kind, and to which mere literary distinction, more especially in foreign countries, can add but little of value. It gives us, however, sincere pleasure to have this opportunity of publicly expressing our sense of Dr. Warren's great merits both as a public and private character, and as a member of our profession.

The frequent occurrence of tumours in every texture of the body renders an acquaintance with them of great importance. The wide difference which exists among them,—the small size, the stationary condition, the harmless and inoffensive nature of some, the magnitude, the rapid progress and the destructive tendency of others, demand an exact discrimination; and a correct diagnosis becomes the more essential as the life of a patient may in some instances depend upon a timely interference; and as the disease—in most cases open to the view,—remains, both before and after death, a permanent token of the sound or incorrect opinion of the surgeon. But, however desirable it may be to distinguish different tumours from each other, to appreciate the degree of danger attendant upon each, and to know whether an operation will be permanently beneficial; the present state of our knowledge, notwithstanding the pains already bestowed upon the subject, does not enable us in all cases to attain this degree of certainty. Ever since the term *malignant* was introduced into surgical nomenclature, as expressing a specific character, it has been a most interesting subject of speculation whether a tumour possess this quality or not; and, indeed, if we take as the grand feature of this class an invariable and irremediable tendency to the destruction of life, the question becomes one of the most serious that a surgeon can be called upon to decide: and yet how greatly have we to regret the uncertainty and confusion that prevail on this point, and how frequently do we find that the most able practical men are altogether at variance, not, indeed, respecting names and definitions merely, or minute and speculative distinctions, but on points of the utmost importance,—the propriety or impropriety of performing severe operations! If the same tumour, even after its removal, be presented to different pathologists, it is not uncommon for one to declare that it is malignant; a second, that it is non-malignant; a third, that its malignity is excessive; a fourth, that it possesses a low degree of malignity; a fifth, that, although it is not yet malignant, at some future time, sooner or later, it may, or it certainly will become so. In cases of this kind, the question which the patient asks is this: "Do you advise me to submit to an operation?" To this it is a rational and no unphilosophical answer to say, "If you do not, this tumour may perchance cause your death." Such, however, is not the reply which a surgeon or pathologist would desire to give: it conveys the acknowledgment of an imperfect acquaintance with the disease. Therefore, while we laud the candour which does not profess greater knowledge than is really possessed, we think it would only be just, at the same time, to admit an equal ignorance as to whether the performance of the operation, malignancy being supposed, will at all retard the fatal period; whether, in fact, the disease be so far local as to receive a material check by the eradication of what has been already developed; or whether it be so far constitutional as to make its progressive advances, notwithstanding its apparent removal, as rapid as if remedial measures had not been adopted. This is a question to settle which requires an improved knowledge of the essential nature of such tumours, and also extensive experience of the effects of operations and of the uninterrupted course of these complaints. We cordially join Dr. Warren in the hope that the investigations of Sir Astley Cooper, whose aptitude and opportunities for the task exceed that of any other man, will, ere long, throw light upon a subject at once so important and so obscure. The two following extracts from the work before us bear on this difficult point: the first relates to scirrhus of the breast; the second to fungus of the testicle.

"Experience has satisfied me that many cases may be cured by operation; according to the best of my information, I should say that one case in three had been cured without relapse. When, therefore, the tumour is not large, has not been of long standing, is not ulcerated, and there are no signs of constitutional affection, it is best to try an experiment, which, notwithstanding its painfulness, is generally desirable and satisfactory to the patient. I wish I could say anything more definite on so important a point, but I am not at present able to do so." (P. 278.) . . . "The statement of these cases will give you an idea of the disposition there is to constitutional disease in the fungoid testis. Whether this constitutional derangement precedes or is consequent on the local malignity, we cannot certainly determine, because we cannot examine the internal organs in an early stage: the common opinion is, that the evil begins in the local affection. Until this point shall be decided in a way contrary to this opinion, it must be considered a matter of the greatest consequence to extirpate the testicle at an early period. But how are we to know whether the disease is of a malignant character before malignant symptoms arise? Simple chronic inflammation, scrofulous and venereal inflammation, may produce appearances similar to the early stage of the fungoid disease. How, then, are we to discriminate the fatal disorganization in season to arrest its transmission to the great organs? It must be admitted that this discrimination is often impossible in the early stage. We must be careful to study the history of the case, and satisfy ourselves that there is not a scrofulous, syphilitic, nor sympathetic action going on in the part. We must enquire if the patient has suffered much pain, whether the functions of the digestive and pulmonary organs are disturbed; we must notice whether the tumour is irregular and comparatively hard. In the case where these symptoms shew themselves, we are not to wait. Better is it to remove a number of testicles without absolute necessity than to allow one to proceed beyond the reach of art. The scrofulous, and even the venereal inflammation, when they have continued long, produce a disorganization from which it is impossible to recover. The organ, in such instances, is of no use; it is but a name; and though men are naturally tenacious of preserving it even in this unsubstantial state, they would not desire to extend the experiment long enough to endanger life. When they are thus determined, it is better that the patient should take this responsibility on himself; and therefore, without exciting unnecessary fears, I should fairly state to him the dangers he has to encounter and let him decide the question.

"Even the scrofulous testis in an ulcerated condition is better removed; for, although the healing of such ulceration is often accomplished, the texture of the part is broken down, as already stated, and its functions lost in many cases. The cure is long, and causes much pain and embarrassment. I have known, and have at this time in my care, a number of patients who have had chronic enlargement of the testicle for many years. I do not propose to remove such tumours, for the following reasons: because the patient suffers no pain, because his health is unimpaired, because the disease does not advance, and because I can trace the origin of these tumours to some satisfactory cause not connected with the character of malignity." (P. 335.)

Whilst, in the main, we consider this to be the statement of a judicious and discriminating practitioner, we cannot allow it to pass without deprecating a mode of expression calculated to lead to the reckless performance of operations. We cannot for a moment suppose that a surgeon of Dr. Warren's experience would undertake the removal of a testicle that was not affected with a disease which rendered it either dangerous or useless; but we look with much apprehension on the establishment of such a maxim as that "*it is better to remove a number of testicles without absolute necessity than to allow one to proceed beyond the reach of art*," lest it should afford a cloak to the ignorance of inattentive or superficial observers. This view of the eligibility of an operation depends on the probability of the disease being malignant; and though it be true that, in the early stages of the disease in question, our present knowledge affords no clearer indication than can be derived from what may happen, or is likely to happen, still the want of certain information should inspire us with extreme caution, and render us unwilling to perform an operation which may subject us to a charge of useless or unnecessary interference. At the same time, it is hardly to be admitted that the total inutility of the organ is quite so probable a consequence of the chronic, scrofulous, or syphilitic inflammation as the preceding account requires us to suppose. The following extracts from Sir Astley Cooper's work on the testis will set this point in a clearer light.

"When I make a section of a chronic enlargement of the testis, throw it into water and agitate it, a whitish yellow fluid proceeds from the seminiferous tubes, which are extremely dilated and which then appear emptied. But still the same bulk of testicle remains, owing to the cellular membrane of the part being loaded with a yellowish fibrine or coagulable lymph; the rete is filled with the same secretion as the tubuli; the epididymis is similarly diseased, and sometimes the vesiculæ seminales and vasa deferentia are distended with a similar morbid secretion. But the effusion whether placed in one situation or in the other, when it becomes absorbed by proper treatment, may and apparently does leave the testicle capable of performing its functions, and allows therefore of its complete recovery." (*On the Testis*, p. 37.)

"At length [after suppuration] the testes diminish, form but a small quantity of semen, and they continue to waste until but a small portion of them remains and their secretion almost entirely ceases. Mr. S. had a scrofulous disease in each epididymis and both testes wasted. Only two or three drops of their fluid issued under an unnaturally excited emission. He had erections and occasionally a desire for sexual intercourse but had no nocturnal emissions. He had the disease four years; and a sinus from each epididymis still discharges a quantity of fluid which stiffens the linen." (*Ibid*. p. 98.)

"When the venereal poison affects the testicle, it probably attacks the tendinous structure, for example the tunica albuginea; and from thence extends into its interior fibrous and not its tubular part; but this I allow to be hypothetical, and am led to that opinion from the structure of that part most resembling the periosteum in its tendinous composition and from the very ready and complete recovery of the organ." (*Ibid*. p. 104.)

When, therefore, we regard the specific function of the testicle, and consider it as an organ giving rise to numerous important sympathies, the tenacity with which men commonly cling to the preservation of it is neither unnatural nor excessive; and we cannot allow that, before the loss of its power or the danger of the complaint be placed beyond doubt, the surgeon is justified in performing extirpation.

There has always been the greatest difficulty in accounting for the origin of tumours: we cannot say that, in the following observations,

Dr. Warren has removed much of the obscurity in which the subject is involved.

"What is the cause of tumours? An individual making a violent effort of some muscle has a tumour formed afterwards in the muscle, and we say the tumour is caused by the strain; that is, the strain changes the healthy action of the capillary exhalants of the part, so that, instead of their usual deposit, they form a morbid substance: or he may receive a blow on the testicle, and a disease succeeding, we say the blow is the cause. A female has a mammary abscess which leaves a chronic induration, and, when she reaches a particular period of life, this assuming the character of a scirrhus, we say the induration is the cause. But is it not notorious that similar accidents occur constantly, and are not usually followed by such results? Of course, it is impossible that those injuries are the simple causes of morbid actions generated in such cases. We must look for accessory causes, such as some peculiarity in the accident or in the individual. The former is inconceivable; and we are therefore compelled to suppose a peculiarity in the constitution of the individual. Such peculiarity may sometimes be fairly suspected, as in the case of a female one of whose parents had been cancerous; or in a man who had abused his health by the use of ardent spirits: but how many instances remain where no such cause can be seen; and how many cases are there where no cause, either predisposing or exciting, comes within the cognizance of our observation! Mr. Hunter suggested the notion that a drop of blood, being accidentally effused, became organized, and assumed a growth independent of the system, and continued to grow till it was limited by some obstacle opposed to it. The objection already instanced has been offered to this opinion,—namely, that effusions of blood take place continually without any such consequence." (P. 4.) . . . "Effusion of lymph, or coagulable lymph, from the capillary vessels, has been considered as a possible cause of the origin of tumours. The lymphatic effusion must of course become organized, and thus constitute a new-formed substance: such effusions are common after accidents and during diseases. We notice them in the cellular texture, in the muscles, in the serous cavities. In the first two of these textures they more rarely become organized, but are soon taken up by the absorbents and disappear; in the last, organization occurs in a great number of instances, but does not produce a tumour. The effusion of lymph, therefore, is not necessarily followed by tumour, even when it becomes organized; yet that some tumours are generated in this way I have no doubt. A third supposable mode of production of tumours is by chronic inflammation of a natural texture. This chronic inflammation happens most frequently in parts disposed to inflame, as the cellular texture. Steatomatous tumours are caused in females, in the cellular texture, on the upper part of the deltoid muscle, by the pressure of their dress, with a frequency that is notable. The greater number of tumours being formed without any such accidents, so far as we know, this chronic inflammation, if admitted to be a cause, is only a partial or occasional one. On the whole, therefore, it must be understood that we have but a very imperfect knowledge of the predisposing and exciting causes of these derangements of organization." (P. 6.)

The list of causes here enumerated cannot be considered as complete; for (not to mention hernial tumours, which, as not being morbid growths, do not fall under notice in this volume,) we find no allusion to parasitical animals forming hydatid tumours, nor to the development of cysts supposed by some to include a numerous and important division, nor to the obstruction of ducts, which as a cause of tumours is far from hypothetical; nor, finally, to the alteration and distension of the coats of vessels, giving rise to the interesting class of aneurismal tumours.

In the present work an exact or scientific arrangement has not been attempted. The plan adopted is that of presenting the different tumours under the head of the different textures of the body, as far as could be done. The author treats first of those which are the most superficial, most easy of inspection, and the affections of which are most simple.

Beginning with the textures of the skin, he next takes those of the cellular membrane; then the muscular, the fibrous, and the osseous. After these most obvious and extensive textures, he places the numerous tumours of the glandular texture; next those of the vascular; and then those of the membranous textures, mucous, serous, fibrous, and synovial. Finally, he notices tumours composed of different textures and not found exclusively in any,—the encysted tumours.

Our space will not permit us to follow the author through the details of this extensive plan: nor do we find a sufficient amount of novelty to tempt us to do so. The cases, however, are all exceedingly illustrative; and we proceed to extract one or two examples which bear upon particular objects of interest. The first we select is one of scirrhus of the breast, and is principally remarkable on account of its termination.

"The recent specimen we have now before us is one you saw amputated, about a month since, by Dr. Hayward. It has some peculiarities: the scirrhous texture involves every part of the breast; the glandular structure and the intervening cellular texture is lost in the new-formed granulated white scirrhous effusion; it is remarkably hard: yet this disease had not been long in coming to the state you see. The patient was a well-formed woman, of thirty-five, rather delicate in appearance, and had never been robust. About a year since she discovered a lump in the right breast, which at first was not painful, but had become so lately, and she had sometimes required opiates at night. The monthly evacuation had been regular to the time she entered the hospital. Two of her father's sisters had been affected with cancer of the breast, and had undergone operations. The disease, so far as she knew, did not return in them; as one died of consumption twenty years after operation, and the other is still living.—This patient, Ann Elkins, went through the operation well, and the following week passed without any unfavorable symptoms, excepting some degree of difficult breathing. In eight or ten days this difficulty increased; the pulse became very quick, the abdomen tumid and painful; and, after three or four days' suffering, she died unexpectedly. A termination so sudden and unlooked for led to a careful examination of the body after death, and the following appearances were noticed:—External appearance of the body natural, excepting some tumefaction of the abdomen. On opening the cavity of the thorax, the lungs were adherent to the parietes very closely on the right side, and slightly on the left. The non-adherent surfaces were reddened, and uniformly covered with a thin fresh layer of lymph. The surface of the pleura exhibited some irregularities, as if there were tubercles within: this appearance was found to be caused by a thickening of the cellular texture under the pleura. The interior of the right lung was in a state of hyperemia, the upper lobe to a great extent; the two others less so, but not natural. The left lung was in a slight degree affected in the same way. The mucous membrane of both lungs, and of the trachea, was very much reddened, and somewhat thickened. The longitudinal fibres were strongly marked in all the large bronchial tubes, as in persons affected with chronic bronchial inflammation. Emphysematous appearances were visible in the lower part of the lungs. The cavities of the heart were quite empty. The foramen ovale was so far open as readily to allow an instrument to pass from the right to the left auricle, through an aperture a quarter of an inch long. In the cavity of the abdomen, the intestines were excessively distended with flatus. The whole peritoneal surface, both lining and investing, was covered with a layer of coagulated lymph, and the capillary vessels injected with red blood. The liver, of a lighter colour than common, was of a crisp consistence, and its coat opaque. The uterus was enlarged, thickened, and very hard: its fundus was covered with a tumour as large as a walnut, excessively hard, so as to be cut open with difficulty. The cut surface presented an arborescent appearance of white striæ, branching from the pedicle which connected it with the body of the organ, exhibiting the pedunculated growth described by Dr. Hodgkin. The right ovarium was increased in size, hard, and contained a number of enlarged ova, some of the size of a pea, with thickened, hard, white coats. The left ovarium was double its common size, soft, and presenting a number of flaccid sacs. Near the ovaria were seen two or three membranous strings, from one to three inches long, with a cyst at the extremity of each lying unattached in the cavity of the abdomen. An incision into the substance of the uterus exhibited two or three hard white nodules, like small pebbles of quartz. The membranes of the brain were not inflamed, and nothing morbid was seen in its substance. The lymphatic glands in the axilla were rather large: one of them had three times the usual size: this gland being divided was found much reddened within, and contained two kinds of structure; an external, of a deep red, and an internal, composed of white rays. Two or three absorbent vessels, running into this gland, were considerably enlarged from their natural size. The cellular texture surrounding and enveloping this gland was intermixed with striæ of white fibres.

"This patient was unmarried, of excellent character, of a constitution not vigorous, but rather inclining to scrofula. No cause was known for the disease. The immediate consequences of the operation were in every respect favorable; the wound was in a healing state when she was seized with fever. The existence of erythema erysipelatosum in the hospital at the time gives us reason to believe that this patient was affected and died with an erysipelatous inflammation of the internal organs. No cause we are acquainted with can so well explain this sudden and unexpected termination. Nor is such an occurrence new to me: I have seen numbers of patients perish a few days after operations, at the time that erysipelas prevailed in the hospital, without the slightest external erythema. In such instances I have been in the habit of stating to you that these patients died of erysipelas, as truly as if they had been covered with an erythematous eruption. The disease is constitutional: it may affect the skin, and generally does so; it may affect the internal organs without affecting the skin, and in such cases is most dangerous." (P. 251.)

The next case we give in an abbreviated form: it is chosen on account of the illustration it affords of the introduction of air into the veins during the performance of an operation; a phenomenon which seems now established by too many instances and too high authority to admit of doubt. No argument, *a priori*, derived from the anatomical study of the heart's cavities, could have led to the supposition that a capability of active expansion resided in its parietes; nor would such an explanation as the author has afforded of the mode in which air gains admission have induced any one to expect such an occurrence, in the absence of direct experience: but it is vain to oppose theory and speculation to repeated and well-attested facts; and, though we still urge a rigorous scrutiny in all such cases as may occur, we feel bound to give credence to observations so carefully made, however extraordinary the relation may appear.

"Nancy Barker, of Trenton, in Maine; married, æt. 33. Three years since she noticed an induration in the right breast, which increased till it involved the whole gland in a tumour, very hard, moveable, yet connected with the pectoral muscle by a morbid adhesion. The nipple drawn in. The axilla is occupied by a considerable tumour, of a globular form, and quite hard. An operation was performed on the 24th of December, 1831. The patient sat in a chair. The right arm was extended and raised above a horizontal line, in order to give tension to the skin and permit access to the armpit. The skin on the surface of the breast, with the diseased nipple, was included in an oval incision. The breast was dissected from the pectoral muscle, and left connected with the axillary glands, while the extirpation of these glands was effected. As they adhered to the great axillary vessels, they were cautiously detached by dissection; and, by insinuating the finger where the cellular substance was loose, between the tumour and the great vein, this separation was nearly effected, only a slight connexion still existing at either extremity of the tumour. Proceeding to separate it, at the outer part of the axilla, a vein was divided, and a small quantity of venous blood discharged. Scarcely was this done when the patient struggled, her complexion changed to a livid colour, and at the same instant a bubbling or gurgling noise, which had not been noticed before, was heard, though indistinctly; but the place from which it issued was not visible, the surrounding skin and fat lying over it. On this, the axilla was immediately compressed. The patient became insensible, breathing as in apoplexy. The tumour was at once separated. The posture of the patient was changed, and stimulants were diligently administered; but the pulse became less distinct every instant, the lividity of the cheeks increased, the body grew cold, and the respiration more and more feeble until it entirely ceased. The lungs were inflated as a last effort, but without avail.

"In order to understand the manner in which air gains admission into the veins two things are to be considered: the state of the heart, and that of the affected vein. First, the heart: This organ has a dilating as well as a contracting power. The auricles, after contracting, dilate by an active motion, and suck the blood from the neighbouring veins: by this suction of the right auricle it is that air may be drawn in at the opening in the wounded vein. Second, the condition of the vein is to be regarded: The coats of the veins are flaccid, and, in their ordinary state, an attempt to suck in air at any aperture would be followed by a collapse of the walls of the vein; and the introduction of air in this way would be impossible: but, if the coats of the vein are prevented from collapsing by an adhesion to an unyielding substance, air might be sucked into and through it without any difficulty. If a suction-hose were composed of a thin skin, water could not be drawn through it; but if it were covered with metal, to which it adhered, there could be no obstacle to the suction process. In order that air may be sucked into a vein in the living body, its coats must be prevented from collapsing; and this may be done by different causes. First, by position: If the arm, for example, be extended to the utmost degree, the axillary vein will at the time be in a state of tension; and, should an aperture be made in it in that situation, the vein could not collapse, and air might be drawn in. Second, if the vein passed through and adhered on the outside to a firm tumour, the vessel could not collapse. Third, the same result would be produced by an attachment to surrounding fascia, which is again attached to bone. Other causes might produce the same effect; as the situation of a short vein between two others, with each of which it is connected: the situation, for example, of the transverse jugular veins, which pass across from the anterior to the middle jugular. When such conditions of the veins near the heart are taken into view, together with the suction-power of the right auricle, there seems to be no difficulty in explaining the entrance of the air into the vein and heart. The mode in which air introduced into the circulatory system causes a derangement of the vital functions is not so satisfactorily explained. The organs whose functions have been supposed to be especially deranged are the lungs, the brain, and the heart. The lungs have been found to contain air in their sanguiferous vessels; and, as the phenomena bear a resemblance to those of asphyxia, this organ has been supposed to be the primary seat of derangement. The livid appearance of the skin, also, and the gurgling noise in the chest, seem to indicate an affection of the lungs; but the latter symptom arises merely from the passage of air through the blood of the heart and of the lungs; the former from the imperfect state of the circulation. The brain, by others, is believed to be affected by the introduction of air into its blood-vessels. Bichât produced appearances in animals similar to those from this accident, by injecting air into the carotid artery; and, in our own cases, the privation of sensibility, the slowness of the pulse, and the heavy respiration, appear to indicate pressure on the brain. It has been said that the time is not sufficient for the imbibed air to reach the brain; but there is a sensible interval between the entrance of the air and occurrence of the phenomena,—an interval quite sufficient for the air to pass through the right side of the heart, the lungs, the left side of the heart, and the carotid arteries, to the brain. In the examinations of the bodies of some of the victims of this accident, air has been found in the right auricle of the heart. This fact, taken in connexion with the slowness or total failure of the pulse, has been thought to prove that the great organ of the circulation is the principal seat of the disorder. The experiments of Nysten favour this explanation, and seem to shew that the distension of the heart by air is sufficient to account for the fatal effects of the introduction of that fluid into the veins." (P. 259.)

In a work exclusively treating of tumours, we are not to be surprised at meeting with the description of some operations which seem to have been undertaken with somewhat too bold a hand, and where the risk which the patient ran of perishing under the knife should rather serve as a warning than as an encouragement to imitation. In this light we are disposed to view the operations performed upon the parotid and thyroid glands; though we willingly concede that Dr. Warren undertook these and other operations aware of the difficulties and dangers, and well prepared with skill and anatomical knowledge to encounter and overcome them. He thus describes the extirpation of the parotid gland:

"An incision, three inches long, was carried from behind the ear to below the angle of the jaw, in a slightly oblique direction from behind forwards. This not affording room, a transverse incision, an inch and a half long, was carried from the anterior lip of the wound in the direction of the lower edge of the jaw, and just below this bone. The tumour was then dissected from the face, ear, and mastoid muscle. The facial or external maxillary artery was cut, and immediately tied. The tumour being disengaged as much as possible from the parts above mentioned, was seized with a double hook at the inferior part, and drawn outwards. The dissection under its lower edge was carried on cautiously until it became deep, and the pulsations of the carotid were quite visible, though the vessel itself could not be discovered so as to secure it. Now being sensible of the probability of involving the external carotid artery, I requested an assistant to compress the common carotid, and then began to pursue the deep dissection. In an instant the assistants, the operator, and the ceiling of the room were covered with a torrent of blood. The common carotid was at once compressed effectually. The blood being sponged off, I sought for the divided artery, but soon found it had retreated below and within the digastric and stylo-hyoid muscles. These muscles were dissected, the artery brought into view and tied, the operation continued and concluded." (P. 288.)

The patient recovered.

Dr. Warren is not deterred by the fears that restrain the operators of this country from dividing and placing ligatures upon veins. For a varicose state of the leg he recommends the division of the vein below the knee, in the following manner: "A bandage being placed above the knee, to prevent the entrance of air, the skin and vein are to be pinched up together, and divided at one cut;" and he remarks, "there is no danger of reunion of the vein; nor have I ever seen any other bad consequences in the many cases in which I have operated by division of the vein." (P. 436.)

Of varicocele a single case is related, in which a ligature was applied with success upon the spermatic veins, (p. 439;) but the operation which is principally recommended for this complaint is the excision of the spermatic veins. This we are told has been done many times with success, and never with any unpleasant consequences, excepting in a single instance: then, however, the consequences were unpleasant in no very slight degree, inasmuch as hemorrhage occurred, and produced an extensive slough of the scrotum, in the separation of which the testicle itself was removed.

"The proper course," says Dr. Warren, "would be, to let the patient know that the testicle is probably useless before the operation, and that after it the organ may wither and shrink away. I mean to say that it may wither up, not that it necessarily will do so. We must then leave to the patient the decision of the question whether he will continue to support the disease or take the consequences of the operation.

On the whole, I consider excision of the veins as the only sure mode; and that it is generally free from any bad consequences which may not be obviated, excepting only the wasting of the testicle." (P. 444.)

The frequent occurrence of varicocele, in a more or less severe form, makes it much to be regretted that the advantages of the operation here proposed should admit of so important an exception; one, indeed, which entirely excludes it from all cases in which the organ is not already entirely useless, or in which the sufferings of the patient are not excessive. When this condition has become such that castration would be justifiable, the operation of tying the veins would also be allowable, and perhaps preferable; but, until the last stage of the disorder has been reached, there will not be found many who would not, on a fair representation of the circumstances, rather content themselves with the palliative treatment, however troublesome and ineffectual.

The histories of Abdominal Tumours, which close the volume, are introduced by some judicious remarks respecting the mode of making an examination, and afford a very clear and simple description of the diagnostic signs which distinguish the varieties of these complaints; but, as we shall have occasion to return to this subject in noticing the cases of Hydatid Tumours of the Abdomen, published by Dr. Bright in the last Number of the Guy's Hospital Reports, we shall here close our remarks on Dr. Warren's book. Of the engravings by which the text is illustrated we may briefly remark, that, although they are not to be compared in point of elegance with those in the papers just referred to, and still less with those in Dr. Bright's former works, yet the praise of general accuracy and usefulness must be awarded them. By augmenting the impression produced by the narrative, they serve to fix on the mind the objects to which they refer, and supply the deficiencies of language where this is inadequate to convey distinctly the appearances of disease.

---

